# An educational guide for nanopore sequencing in the classroom

**DOI:** 10.1371/journal.pcbi.1007314

**Published:** 2020-01-23

**Authors:** Alex N. Salazar, Franklin L. Nobrega, Christine Anyansi, Cristian Aparicio-Maldonado, Ana Rita Costa, Anna C. Haagsma, Anwar Hiralal, Ahmed Mahfouz, Rebecca E. McKenzie, Teunke van Rossum, Stan J. J. Brouns, Thomas Abeel

**Affiliations:** 1 Delft Bioinformatics Laboratory, Delft University of Technology, Delft, Netherlands; 2 Kavli Institute of Nanoscience, Department of Bionanoscience, Delft University of Technology, Delft, Netherlands; 3 Broad Institute of MIT and Harvard, Boston, Massachusetts, United States of America; 4 Leiden Computational Biology center, Leiden University Medical Center, Leiden, Netherlands; University of Toronto, CANADA

## Abstract

The last decade has witnessed a remarkable increase in our ability to measure genetic information. Advancements of sequencing technologies are challenging the existing methods of data storage and analysis. While methods to cope with the data deluge are progressing, many biologists have lagged behind due to the fast pace of computational advancements and tools available to address their scientific questions. Future generations of biologists must be more computationally aware and capable. This means they should be trained to give them the computational skills to keep pace with technological developments. Here, we propose a model that bridges experimental and bioinformatics concepts using the Oxford Nanopore Technologies (ONT) sequencing platform. We provide both a guide to begin to empower the new generation of educators, scientists, and students in performing long-read assembly of bacterial and bacteriophage genomes and a standalone virtual machine containing all the required software and learning materials for the course.

## Author summary

Genomes contain all the information required for an organism to function. Understanding the genome sequence is often the key to answer important biological questions. For example, the sequences of human genomes are used for diagnosis of genetic disorders or for the development of personalized treatments, while the sequences of microbes may inform about their mechanisms of infection and guide the development of novel drugs. Today, our capacity to generate genome sequencing data is tremendous. However, our capacity to process this information is insufficient. This is partially due to limitations of current methods for data analysis but is mostly caused by lack of training for most biologists to leverage high-throughput sequencing data and use their full potential. It is urgent that we train the new generations of biologists to become computationally aware and able to keep pace with technological developments in the field. In this manuscript, we illustrate our efforts in adopting an integrated teaching model that bridges experimental and bioinformatics works. Our course integrates data generation in the lab with bioinformatics work to illustrate the interlinking of lab practices and downstream effects. In our demonstration course, we used nanopore sequencing to train nanobiology students, but the model is easily customizable to suit students of different educational backgrounds or alternative technologies. The tools we provide help not only science educators but also biologists to address many relevant questions in biology.

## Introduction

What defines a biologist? In short, a biologist is a person who studies life and living organisms. But this simple definition hides the true complexity of the field of biology. Biology covers diverse topics such as molecular biology, structural biology, ecology, evolution, genetics, microbiology, immunology, and biotechnology. Importantly, most (if not all) of these topics have undergone incredible progress due to rapid discoveries and technological advances[1,2]. As such, a modern biologist has the inevitable tasks of adapting to rapid change and mastering new knowledge and technology.

One of the most important revolutions in the field of biology was caused by the development of next-generation sequencing (NGS) technologies. Using massively parallel processing of samples, NGS dramatically reduces sequencing time and costs, enabling the sequencing of entire genomes. Currently, genome sequencing and analysis have become a crucial component in biology, as evidenced by recent scientific breakthroughs [3,4] and by the exponential increase of reported genomes on GenBank (e.g., from 30,000 sequenced prokaryotic genomes in 2014 [5] to 183,000 in 2018 [https://www.ncbi.nlm.nih.gov/genome/browse/#!/overview/], a 6-fold increase in only 4 years). Thus, not only do biologists need to adapt and learn how to use these emerging technologies, they also need to learn how to mine the ever-growing mountain of genomic information they generate, which requires bioinformatics skills. Now, the question is how do we train this generation of biologists so that they have the required computational skills?

## Bridging bioinformatics to biologists

Over the past few years, we have taught introductory bioinformatics to undergraduate (second year BSc) biology students with basic molecular biology training. They are versed in standard techniques (such as basic DNA extractions and PCR) but are unfamiliar with specific DNA sequencing chemistries. In the past, this mandatory computational course was entirely disconnected from lab work, making it hard for students to grasp how bioinformatics and biology are connected. To address this disconnect, we here share a more integrated approach to teach bioinformatics to biology students. These students have a conceptual grasp of sequencing and bioinformatics but not the detailed view on how various lab techniques (e.g., NGS chemistries) combined with various analysis methods (e.g., assembly, variant calling) can be used to answer specific biological questions and how these techniques interact with each other.

**Fig 1:**
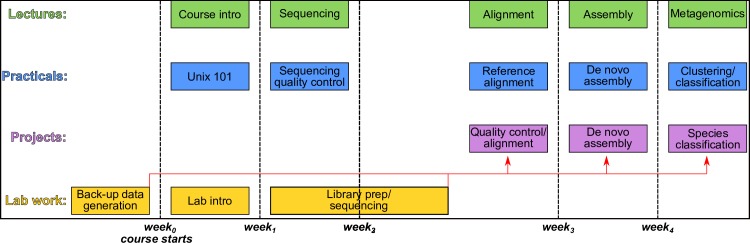
Course overview. Integrated bioinformatics training with time on the x-axis. Lectures (green) give students the necessary background to execute and understand Practical (blue) and Project (purple) sessions. Laboratory sessions (yellow) enable students to employ their biological background and prepare their own DNA libraries from samples of interest. Libraries prepared by each student group are pooled together and run on a MinION device (Oxford Nanopore Technologies, Oxford, UK), generating data to be processed in Project sessions. Backup data previously prepared from the same samples can be used if the students’ MinION run fails to provide enough quality data for analysis. In the Practical sessions, students learn to use established bioinformatics methods, with an emphasis on processing long-read data (see Fig 2, S1 Table and S1 Text). In the Project sessions, they then apply these methods to the generated data to answer specific research questions. After intragroup and intergroup discussions of results, students prepare their final project report and present their results in a poster format.

The overall idea is to start from where students are already familiar (i.e., biology) and expand from there. There are 4 types of learning activities in the course (see Fig 1): (1) lectures in which students receive classroom instruction on bioinformatics topics, (2) practical sessions in which students apply the material from the lectures to solve practical exercises supervised by teaching assistants, (3) lab work in which sequencing data are generated, and (4) a project that applies the bioinformatics concepts learned in the lectures on data from the lab work. This is concluded by a poster session in which all students get to review each other’s work. A week by week overview can be found in S1 Table.

The formula presented here focuses on introducing bioinformatics to biology students, helping them to acquire the skills and insights needed to operate and troubleshoot existing algorithms. The course does not focus on developing skills needed to create novel algorithms or models.

During the pilot run of this course in the academic year from 2017 to 2018, we used Oxford Nanopore Technologies (ONT) MinION sequencing as a data generation platform. This platform was selected because it has low capital cost and is a new exciting technology easy to engage students with. Real-time data acquisition gives immediate feedback to the students that data are being produced, even if they have to keep it running overnight. It is easy to imagine they could get one of these devices at home. Students can see themselves as scientists, as people discovering something new, an idea that we really like to foster. Ultimately, any fast, cheap, and accessible sequencing platform would be good for our goals, yet only MinION is currently available.

MinION has already made its way into undergraduate and graduate courses [6,7]. Some of these courses focused on data analysis; they organized hackathons in which students needed to devise a pipeline to infer the ingredients of food DNA samples or identify human DNA samples[6]. Others developed the application of MinION further by also teaching laboratory techniques for DNA extraction and sequencing library preparation[7].

Additionally, the portable size of ONT’s MinION and the simplicity of library preparation enable scientists to use this technology in a wide variety of environments, including a standard classroom[8–10]. As such, this device is not only attractive for researchers but also for educational instructors: If this technology is empowering scientists to embark on novel scientific studies, why not also empower young students to embark on effective educational experiences?

## Integrating nanopore sequencing in the classroom

The challenge set for students in our course was to identify and discover novel phages from environmental samples and to reconstruct complete genomes from single-isolate and metagenomics samples. The students had to address the following research questions, which were introduced at the very beginning of the course: (1) Can we assemble and annotate fully closed genomes from a small number of long reads? (2) What are the considerations for the assembly of metagenomics samples compared to single isolates? (3) What is the advantage of long-read sequencing for the analysis of metagenomics samples? (4) Can we identify virulent and temperate phages in metagenomics samples? (5) What genes of interest can we find in both bacteria and phage genomes?

Twenty-four groups of 4 students (96 total) prepared their own DNA libraries of various single-isolate bacterial, bacteriophage, and metagenomic samples in the classroom. Number of groups and their size were determined to allow for sufficient supervision within the available lab space. If possible, smaller groups are preferable to increase the hands-on time of each student. We would like to emphasize the benefits of having multiple groups working on different related samples (e.g., each barcode represents a similar but different microbial isolate). This allows groups to initiate discussions about differences in their own findings—such as unique sequences, structural variants and presence and/or absence of genes—and hypothesize how those differences may influence the phenotypic traits of their sample. This exercise helps them further appreciate the value of bioinformatics skills in a biological setting and how the 2 are ultimately connected.

The DNA libraries were prepared using the rapid barcoding kit (SQK-RBK004), which has fewer steps than other available kits and thus allows the procedure to be completed within the 3-hour timeframe of the class. For longer sessions, the ligation sequencing kit (SQK-LSK109) could be used, increasing the robustness and throughput of the experiment. Both kits allow for barcoding of multiple genomic DNA samples. Samples were prepared individually by each group and then barcoded and pooled together at different proportions depending on the success of each group. When sequencing runs failed, the student was supplied with previously generated backup data.

**Fig 2:**
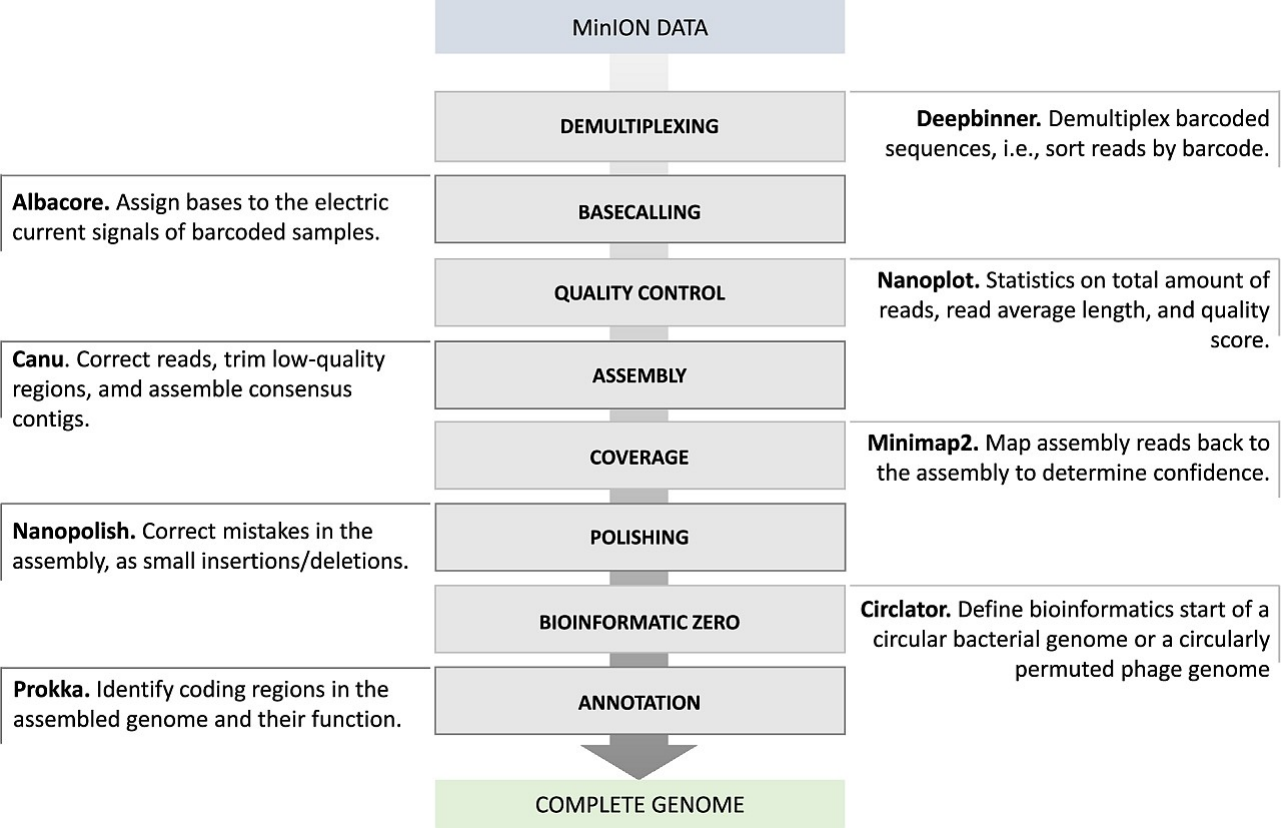
Pipeline for genome assembly using MinION data. First, the barcoded sequences are demultiplexed using Deepbinner[11] and basecalled using Albacore (Oxford Nanopore Technologies, Oxford, UK). Nanoplot [12] is used to assess the quality of the sequencing data for downstream processing. If the data have sufficient quality, they are used for assembly using, e.g., Canu [13]. Confidence on the resulting consensus assembly is obtained using Minimap2[14]. The assembly is polished to remove common mistakes using Nanopolish[15], and then Circlator [16] is used to determine the zero-based start of the genome, which depends on whether it is a bacterial sequence or a bacteriophage sequence. Finally, the assembled genome is annotated using Prokka [17]. Please refer to S1 Text for further details.

After running DNA samples in MinION, students performed quality control of their data and then assembled the genomes. As we focused on teaching technical concepts of bioinformatics, we provided a computational guide (see S1 Text and summary in Fig 2) containing ready-to-go commands and scripts for commonly performed tasks that can be broadly used with MinION data. To facilitate the use of this guide, we provided a standalone virtual machine containing all required software used in S1 Text.

Once data processing was completed, students pursued a variety of research questions, such as investigating the genomic composition of their bacterial sample as well as the population composition of their metagenomics sample. For example, students would determine the bacteriophage species in their barcoded sample and compare their assembled genome to that of the closest reference genome found in the National Center for Biotechnology Information (NCBI) reference sequencing database (RefSeq). In all cases, students found that their assembly had little overlap with the reference, prompting discussions about the novelty of the genetic content in their phage.

Students ran Centrifuge [18], a species classification and quantification tool, on their metagenomics sample and generally concluded a mixture of viral and bacterial species. This process stimulated discussion about a number of course-related topics: (1) limitations of k-mer-based tools (e.g., k-mers are not always unique to individual species), (2) biases when comparing against a reference data set (e.g., you can only classify what you have previously observed), (3) understanding bacteriophage biology (e.g., phages can integrate their DNA in a bacterial host; therefore, sequences that are labeled as “bacteria” may actually correspond to integrated phage DNA), and (4) understanding whether long-read sequencing is advantageous to the scientific question addressed (e.g., long-read sequencing helps improve assembly quality of metagenomes, but the high error rates of the technology still limit its usefulness; here, combining short-read and long-read data could be the best approach to improved contiguity and base pair–level accuracy). These topics were framed to explore how they may affect the student’s computational observations.

## Impact of integrated bioinformatics education

Through the integrated approach in our course, students can easily grasp the direct influence of the experimental protocol on data quality. For example, a student’s excessive pipetting leads to observably shorter read-length distributions, resulting in fewer unique overlaps in the pairwise alignments, a less contiguous assembly graph, and ultimately more fragmented assemblies. Furthermore, the setup is sufficiently generic that different scientific questions could be addressed using this pipeline, and it is sufficiently flexible to adjust to the students’ background.

We experienced increased interest and engagement in our course from both the instructors and the students. Students were much more interested in the course content because they could assume scientific responsibility and ownership. Spending several hours or days in the lab goes a long way to make “scientists-to-be” feel “this is my data.”

The instructors leveraged the practical classes as an opportunity to generate and analyze data for potential pilot studies, i.e., preliminary data for the next round of grants. In our pilot version of the course, the experiments were chosen such that they contribute to ongoing research in the lab. As a result, we generated several follow-up project ideas, one of which resulted in a master’s thesis on heterogeneity of bacteriophage genomes detected by nanopore sequencing, as well as a tripling of the number of undergraduate lab-rotations in the area of bioinformatics.

Naturally, many of the assignments, including interpretation and comparison of a genome assembly from single bacterial isolates to that of viral samples, were open-ended and initially challenged the students. However, the experience gave them a more realistic impression of academic research and foundational skills to help them in their future career as modern biologists. In particular, different samples required different data interpretations, naturally spurring discussions and collaborations among students. Future editions of such an integrated course could consider even developing the student ownership further by explaining the “problem” and asking students to design the DNA sequencing experiments given the boundaries of the reagents available. With adequate supervision and coaching to include proper controls and experiments, this could lead to even greater collaboration and ownership by the students.

## Conclusion

Considering the fast pace at which sequencing technologies progress and at which genomics data are generated, it is no longer possible to ignore the urgency of equipping young biologists with the required skills to manage the amount and type of sequencing data being generated. Here, we used nanopore sequencing as one possible tool to prepare a new generation of bioinformatics-aware modern biologists. Nanopore sequencing offers an exciting opportunity to not only introduce students to the field of genomics and bioinformatics but also to address advanced biological and computational problems. Simple customizations of the assignments are possible to make the course different every year and to make it suitable for teaching students of different backgrounds, such as computer science (e.g., toolbox handling, algorithm understanding), molecular biology (e.g., genomics, sequencing), or medicine (e.g., pathogen detection, cancer diagnostics). MinION also gives a chance to teach the students how to use different tools and community-based analysis and the importance of constantly updating their knowledge of recent technological developments.

The virtual machine and guide provided herein intend to assist science educators and also geneticists to address timely questions in biology, such as detection of epigenetic modifications, characterization of human genetic variation, real-time detection of pathogens, characterization of structural variation in cancer, and analysis of population transcriptomics.

A walkthrough of ONTassembly of prokaryotic genomes and their viruses is provided in S1 Text. All materials, including the virtual machine image, are available at https://github.com/AbeelLab/integrated_bioinformatics.

## Supporting information

S1 Table Detailed syllabus. Detailed overview of course activities week by week. Lecture topics, practical topics, and project work align.
(DOCX)

S1 Text Student walkthrough. Complete student manual with all work to be performed by students.
(DOCX)
