# Supplementary material for: An educational guide for nanopore sequencing in the classroom
Source: PLoS Comput Biol. 2020 Jan 23;16(1):e1007314. doi: 10.1371/journal.pcbi.1007314 (PMC6977714; doi:10.1371/journal.pcbi.1007314)
Supplement: S1 Table — Detailed overview of course activities week by week. Lecture topics, practical topics, and project work align. (DOCX) [file pcbi.1007314.s001.docx]

| Week | Lecture  (morning 2 hours) | Practical  (morning 2 hours) | Lab/Project  (afternoon 4 hours) |
| --- | --- | --- | --- |
| 1 | Course setup  Introduction to bioinformatics  Introduction to biological questions to be answered through coursework | Unix 101 tutorial | Python 101 tutorial |
| 2 | Phylogenetics | Phylogenetics exercises | Lab instruction |
| 3 | Lab work: groups of 4 students, spread over 3 days, total of 24 groups | | |
| 4 | (Whole genome) alignment | (Whole genome) alignment exercises | (Whole genome) alignment in the context of the data generated |
| 5 | Genome assembly | Genome assembly | Genome assembly in context of student data |
| 6 | Midterms | | |
| 7 | Short-read alignment | Short-read alignment | Short-read alignment of public data combined with student data |
| 8 | Clustering/classification | Clustering/classification | Apply clustering to find sub-populations |
| 9 | Hidden Markov Models | Hidden Markov Models | Prepare report/poster |
| 10 | Master class (graduate students present on their related research) |  | Poster presentations and evaluations of all groups. |
