# Supplementary material for: An educational guide for nanopore sequencing in the classroom
Source: PLoS Comput Biol. 2020 Jan 23;16(1):e1007314. doi: 10.1371/journal.pcbi.1007314 (PMC6977714; doi:10.1371/journal.pcbi.1007314)
Supplement: S1 Text — Complete student manual with all work to be performed by students. (DOCX) [file pcbi.1007314.s002.docx]

**Walkthrough of Oxford Nanopore assembly of prokaryotic genomes and their viruses**

Contents

[Required software 3](#_Toc1134681)

[Quality control 5](#_Toc1134682)

[*De novo* assembly with *Canu* 8](#_Toc1134683)

[Calculating coverage 10](#_Toc1134684)

[Polishing the assembly 11](#_Toc1134685)

[Zero-based start of a genome 13](#_Toc1134686)

[Annotating your genome with Prokka 17](#_Toc1134687)

[References 18](#_Toc1134688)

# Required software

For the analysis of Nanopore sequencing data you will need to install the software described in Table S1. You can use our **Virtual Machine** (running Ubuntu 64-bit) containing all software, which can be downloaded at:

<https://github.com/AbeelLab/integrated_bioinformatics>.

Note that this guide assumes that you are familiar with a unix-based operating system (e.g. Linux distributions, macOS). It also assumes your data is basecalled via either Albacore or Guppy (see ONT’s basecalling software).

Table S1. Summary of software required for analysing Nanopore sequencing data

| **Tool** | **Description** | **Source** |
| --- | --- | --- |
| **Required** |  |  |
| *Python* | Programming language | <https://www.python.org/> |
| *Nanopolot* | Package for visualizing long-read data | <https://github.com/wdecoster/NanoPlot> |
| *NanoFilt* | Quality filter for long-read data | <https://github.com/wdecoster/nanofilt> |
| *Canu* | Long-read *de novo* genome assembler | <https://github.com/marbl/canu> |
| *Nanopolish* | Error correction for nanopore-based *de novo* genome assemblies | <https://github.com/jts/nanopolish> |
| *Minimap2* | Long-read aligner | <https://github.com/lh3/minimap2> |
| *Samtools* | Tools for analysing read-alignments | <http://samtools.sourceforge.net/> |
| *Mummer* | Whole-genome aligner | <https://github.com/mummer4/mummer> |
| **Optional** |  |  |
| *Porechop* | Nanopore read adapter trimmer (officially unsupported since Oct. 2018) | <https://github.com/rrwick/Porechop> |
| *Circlator* | Tool for circularize genome assemblies | <https://github.com/sanger-pathogens/circlator> |
| *Prokka* | Prokaryote genome assembly annotation | <https://github.com/tseemann/prokka> |

Alternatively, most of the tools in Table S1 can be installed by using the package manager, *miniconda3* (note: albacore requires a community login and compiling). To use *miniconda3:*

1. Install *miniconda3* by following the [installation instructions.](https://conda.io/miniconda.html)
2. Locate the *miniconda3/bin/* folder
3. Add the */bin/* folder to your PATH variable using the following command:

~$ export PATH=:/path/to/minconda3/bin/:$PATH

1. Set up the following channels **in this order**:

~$ conda config –-add channels defaults

~$ conda config --add channels R

~$ conda config --add channels bioconda

~$ conda config --add channels conda-forge

1. To install all program and their dependencies, run these commands:

~$ conda install -c bioconda canu

~$ conda install -c bioconda samtools

~$ conda install -c bioconda nanoplot

~$ conda install -c bioconda minimap2

~$ conda install -c bioconda porechop

~$ conda install -c bioconda nanofilt

~$ conda install -c bioconda nanopolish

~$ conda install -c bioconda prokka

~$ conda install -c bioconda filtlong

~$ conda install -c bioconda python

~$ conda install -c bioconda deepbinner

~$ conda install -c bioconda circulator

~$ conda install -c bioconda mummer

Note that, if you run into problems with your environment variables, either (1) consider installing the program separately (without miniconda3 and thus compiling from github) or (2) create a separate environment for this program (<https://conda.io/docs/user-guide/tasks/manage-environments.html>).

# Quality control

Quality control (QC) is essential to understand if the run performed well and if the reads are of sufficient quality, length and throughput for downstream processing. ***NanoPlot*** can be used for some statistics. The input for *NanoPlot* is the “sequencing_summary.txt” file created by *Albacore*, ONT’s basecaller. To run *Nanoplot:*

1. Create a workspace directory and run *Nanoplot* with the following command.

~$ mkdir ~/workspace/nanoplot

~$ NanoPlot -t 1 \

--summary ~/workspace/basecalling/sequencing_summary.txt \

-p barcodeXX -o ~/workspace/nanoplot --barcoded

Table S3 below gives an explanation of *Nanoplot’s* parameters.

**Table S3. Description of *Nanoplot* parameters**

| **Parameter** | **Description** |
| --- | --- |
| -t | How many threads you want to use. If you are not sure how many your machine can handle, just use 1 |
| --summary | Path to the summary.txt file. If you have not moved it, it is located in the path specified |
| -p | Prefix to the name of all output files. This can be changed to any name |
| -o | Output directory |
| --barcoded | Performs separate analysis for each barcoded sequence |
| --minlength | Omit reads below this given length |

Note that you can use the “—minlength” and specify an integer to omit reads below the specified length from the analysis.

1. Locate the output folder on your computer. Several plots are generated for you to inspect. Use the figures and the **NanoStats.txt and NanoPlot-report.html** files in this folder to determine what your average read length is, the number of total reads, what your average quality score is and how many of the total demultiplexed fast5 files were of your own barcoded sample. Use the total throughput in bp to **calculate the theoretical coverage** of your expected genome size. Additionally, you can also see the name of the longest reads of your sample. Take these reads and blast them to see what they are.
2. It is worthwhile to remove low quality reads and small reads from your data for downstream processing steps, such as determining contig quality and polishing. Therefore, we use a filter program called ***NanoFilt***:

~$ cat ~/workspace/barcodeXX_raw.fastq | NanoFilt -l 1000 \

-s ~/workspace/basecalling/sequencing_summary.txt \

> barcodeXX_filtered.fastq

Here we are combining two commands with the ‘|’ character. Note that in the second command, the “-l” is used to filter for minimal read length. Oxford Nanopore sequence uses the *phred* quality scoring. *Nanofilt* filters for a quality score (q) above 7 (q = 10 × log_10_ (p), where *p* is the estimated error probability for the basecall) [[1](#_ENREF_1)].

1. Now trim the adapter sequences. During the library precreation, barcoded adapter sequences were added to the DNA fragments. These are not from your organism, so they need to be removed. For trimming, we use ***PoreChop***.

~$ porechop -i ~/workspace/barcodeXX_filtered.fastq \

-o barcodeXX_trimmed_filtered.fastq

1. In the final step prior to assembly, we need to calculate the expected theoretical coverage. For a proper assembly with *Canu*, you will need a theoretical coverage of at least 30X. For smaller (phage) genomes a tenfold increase in the target coverage can be expected. To save computational time, you could take a subset of the reads to an estimated 50X coverage. An easy way to do this is with the program ***FiltLong***:

~$ filtlong ~/workspace/barcodeXX_filtered.fastq \

--target_bases 5000000 > barcodeXX_final_input.fastq

Note that “--target_bases” is used to specify the number of bases you want after filtering. Use the number of bases that correspond to 50x theoretical coverage (*i.e.*, for a phage genome that is 100kb long, 5 million bases amount to 50X coverage).

# *De novo* assembly with *Canu*

***Canu*** is a long-read assembler. It performs three main steps: (1) correction, where reads are overlapped based on alignment, which is used to correct the *read*, based on consensus; (2) trimming, where low-quality consensus regions of the corrected reads are trimmed; and (3) assembly, where overlap-layout-consensus is performed for a final assembly. This will create consensus contigs and a graph of (possible) alternative genome paths (architecture). Prior to assembly, please consider that **you need (at least) four threads** available on your computer.

1. Assemble your genome:

~$ canu -p your_organism_name -d ~/workspace/canu_assembly \ genomeSize=100k maxThreads=4 \

-nanopore-raw ~/workspace/barcodeXX_final_input.fastq

Table S4 below gives an explanation of *Canu’s* parameters.

**Table S4. Description of *Canu* parameters**

| **Parameter** | **Description** |
| --- | --- |
| -p | Prefix name to all main output files. Generally the name of your organism |
| -d | Directory to store assembly results and intermediate information |
| genomeSize= | Approximate size of your organism’s genome |
| maxThreads | Number of threads to use. Note that, although 4 is the minimum required, 8 is recommended |
| -nanopore-raw | Inform Canu that the input data is raw nanopre data. It should be followed by the path to your input reads |

Note: *Canu* expects a coverage of ≈40-50x. If your expected coverage is below 30X, add this parameter correctedErrorRate=0.16. Similarly, if your expected coverage is above 60X, add this parameter correctedErrorRate=0.12

The output will be in the “canu_assembly” folder. Inspect the “.report” file to see how the run performed. You should look at the number of reads used as input, the number of reads used for the final assembly, number of contigs created, and the final assembly size and N50 (weighted median statistic such that 50% of the entire assembly is contained in contigs or scaffolds equal to or larger than this value). The “your_organism_name contigs.fasta” file contains the final assembly contigs in FASTA format. Use the BLASTN web tool to blast your contigs (if they are too large, blast them in segments). The “your_organism_name.contigs.gfa” file shows the paths of all contigs. Open the gfa file in *Bandage* (download to your own device: <https://rrwick.github.io/Bandage/>). Check the number of contigs obtained, if they are linear or circular, and if they have alternative genome paths.

# Calculating coverage

To determine confidence in the assembly output of *Canu*, look at the coverage across the assembly (how many reads represent each position of the assembly). To do this, map the assembly reads back to the assembly. Use a special long read mapper to do this, ***minimap2***.

1. Copy your assembly to your workspace and change directory:

~$ cp ~/workspace/canu_assembly/your_organism_name.contigs.fasta

~$ cd ~/workspace/

1. Align the reads to your assembly:

~$ minimap2 -ax map-ont your_organism_name.contigs.fasta \ barcodeXX_final_input.fastq > alignment.sam

Note that the “-ax map-ont is the pre-set used for Nanopore raw reads. The input is a target sequence (in this case: the assembly) and a query (in this case, your reads). The output is the alignment.sam file.

1. Create a sorted and indexed bam file, which is easier to use in any alignment viewer. For this, use ***samtools***:

~$ samtools sort alignment.sam > alignment_sorted.bam

~$ samtools index alignment_sorted.bam

You now have an assembly (FASTA), sorted alignment file (BAM) and its index (alignment_sorted.bam.bai). We can use these to determine the coverage across the genome. Go to <http://software.broadinstitute.org/software/igv/download> and download the “**IGV webstart**” version of the IGV viewer. Use “file>load from file” to load the bam file. Use “genomes> load genomes from file to” load your FASTA file of your assembly. At the top, select your largest contig. Check if genome is evenly covered by reads. Click on “view>preferences…alignments” and check the “show soft clipped bases” box. Verify how many bases are soft clipped at the start/end of the assembly.

# Polishing the assembly

Nanopore sequencing has a very high error rate (≈10%), which means that reads generated during sequencing are hard to use for annotation. *Canu* corrects many mistakes in the final consensus, but there are often still many small insertions or deletions present in the assembly output. To correct for this, ***Nanopolish*** can be used. This tool uses a signal level algorithm to correct for these common mistakes. If you want to annotate your genome properly, this tool is highly recommended (it is also possible to polish with Illumina data).

1. Index the reads:

~$ nanopolish index \

-d ~/workspace/demultiplexed_fast5/barcodeXX \ ~/workspace/barcodeXX_final_input.fastq --verbose \

-s ~/workspace/basecalling/sequencing_summary.txt

Note that “-d” sets the parent folder of all fast5 files, change this to your barcode. The input are the FASTQ reads. “--verbose" is used to display what is happening on the terminal screen. “-s” is used to specify the sequencing_summary.txt (this will greatly speed up polishing).

1. Start the (time-consuming) polishing step. To speed up polishing, you will split your genome up into several shorter segments with a python script:

~$ mkdir ~/workspace/nanopolish

~$ python /home/nanobiology/miniconda3/bin/nanopolish_makerange.py \ ~/workspace/your_organism_name.contigs.fasta \

| parallel –results nanopolish.results -P 2 \

nanopolish variants \

--consensus polished.{1}.fa -w {1} \

--reads ~/workspace/barcodeXX_final_input.fastq \

--bam ~/workspace/alignment_sorted.bam \

--genome ~/workspace/your_organism_name.contigs.fasta \

-t 1

Note that “nanopolish_makerange.py” is the script used for segmenting the assembly. We will run two segments in parallel with “-P”, although you can do more if you have more threads available. We then pass the output to “nanopolish variants” which is used to polish the genome. Note that, “{1}”, is each 50 kb segment called by the python script, and specifies that we are running the consensus calling mode. Briefly:

**Table S5. Description of *Nanopolish* parameters**

| **Parameter** | **Description** |
| --- | --- |
| --consensus | Output file names |
| -w | Window of polishing, which is the value for {1}. |
| --bam | Path to alignment file in BAM format. |
| --reads | Path to reads file. |
| --genome | Path to assembly file. |
| -t | Number of threads. |

1. Every segment is now polished separately. To **merge** the polished assembly, we use the **nanopolish_merge python script**:

~$ python / home/nanobiology/miniconda3/bin/nanopolish_merge.py \ ~/workspace/nanopolish/polished.*.fa \

> nanopolished_your_organism_name.fasta

~$ cp ~/workspace/nanopolished_your_organism_name.fasta ~/workspace/

It could be useful to rename your contigs for further use. You can do that in ***Notepad++*** or in the terminal itself using ***nano*** (<https://wiki.gentoo.org/wiki/Nano/Basics_Guide>). After renaming, proceed to the next step.

# Zero-based start of a genome

Bacterial chromosomes are circular, *i.e.*, there is no clear beginning or end of the DNA molecule. However, their genomes are always represented in a linear FASTA format. Therefore, we artificially “break” a genome at a certain location, representing each newly acquired genome linearly. Phages also have circular genomes during replication inside their bacterial host, but a phage chromosome is cut and linear inside its capsid. Depending on the exact mechanism of viral DNA packaging, a phage genome assembly can either appear to be circularly permuted or linear. Depending on whether you have a bacterial assembly, a circularly permuted phage assembly or a linear phage assembly, the **bioinformatic zero will be different.**

For **bacterial genomes**, a closed genome is always represented by a large, circular contig. Smaller contigs that are present in the assembly are often viral sequences or plasmids. The bioinformatic start of the contig is most often determined by the *dnaA* gene. This gene plays a key role in the initiation and regulation of chromosomal replication. ***Circlator*** uses a set of *dnaA* genes to find the closest homolog in your assembly with the prodigal gene predicter, and breaks the contig at the start of this gene. It also trims overhangs of your assembly, which are often created by *Canu* output of circular genomes.

1. Create a new folder in your workspace folder and run *Circlator* on your assembly:

~$ mkdir ~/workspace/circlator_output

~$ circlator all --threads 4 –verbose --data_type nanopore-corrected \

~/workspace/canu_assembly/your_organism_name.contigs.fasta \ ~/workspace/canu_assembly/*.correctedReads.fasta.gz \ ~/workspace/circlator_output

Note that “all“ uses all suites of *Circlator*, including fixing the start and trimming overhangs. As input, give your *Canu* assembly (your_organism_name.contigs.fasta) and the corrected reads that are output by *Canu* (your_organism_name.contigs.correctedReads.fasta.gz). Also, “--data_type” is used to specify the input reads, in our case they are nanopore corrected. In most bacterial cases, the dnaA gene is used to start the genome, which is the standard gene searched for by *Circlator*.

1. When finished, you can open the *Circlator* output folder. Notice that there are many files created. Open “**04.merge.circularise.log”** to check if your chromosomal contig was circularized (last column). Open the **“06.fixstart.log”** to check at what gene and at what position each contig is re-oriented. Open “**06.fixstart.fasta**” to check the new contig. Assess how many bases were trimmed off during *Canu* assembly, and if smaller contigs have been re-oriented and trimmed.
2. Copy your new newly fixed start assembly to your workspace folder and go to the next step.

~$ cp ~/workspace/circlator_output/06.fixstart.fasta ~/workspace/

~$ cd ~/workspace/

~$ mv 06.fixstart.fasta final_your_organism_name.fasta

If you have a **circularly permuted phage** genome, the bioinformatic zero is determined based on homology to the small terminase subunit. This protein, together with the large terminase subunit, is involved in packaging of the genome. A FASTA file with small terminase subunits from different phages will be provided to you. You can use this as input in *Circlator*. This program can use the input genes you specify to break the assembly at the start of a closely related homolog, using the prodigal gene predicter. It also trims overhangs of your assembly, which are often created by *Canu* output of circular genomes.

1. Create a new folder in your workspace folder and run *Circlator* on your assembly:

~$ mkdir ~/workspace/circlator_output

~$ circlator all --threads 4 --verbose \ ~/workspace/canu_assembly/your_organism_name.contigs.fasta \ ~/workspace/canu_assembly/*.correctedReads.fasta.gz \ ~/workspace/circlator_output \

--genes_fa ~/workspace/small_subunit_terminases.fa

Note that *“--genes_fa”* is used to give a list of genes to search for instead of the standard *dnaA* gene, in our case a list of small subunit terminases. This gene will be the starting gene of the genome.

1. When finished, you can open the Circlator output folder. Notice that there are many files created. Open “04.merge.circularise.log” to check if your chromosomal contig was circularized (last column). Open the “06.fixstart.log” to check at what gene and at what position each contig is re-oriented. If the gene name is “prodigal”, then it did not find a homolog and started the genome at a random gene. You might be able to manually fix this after annotation (next step). Open “06.fixstart.fasta” to check the new contig. Assess how many bases were trimmed off the original *Canu* assembly.
2. Copy your newly fixed start assembly to your workspace folder and go to the next step.

~$ cp ~/workspace/circlator_output/06.fixstart.fasta ~/workspace/

~$ cd ~/workspace/

~$ mv 06.fixstart.fasta final_your_organism_name.fasta

**Linear phage assemblies** are, when correct, the actual representations of the chromosome within the virion. No bioinformatic zeroing is thus required. However, these linear chromosomes can have repeats at their ends, termed **direct terminal repeats**. We can trim the repeat on one end, by aligning the assembly against itself, with ***Nucmer***. *Nucmer* is a tool that can pairwise align sequences based from anchoring matches.

1. Assuming you have one contig:

~$ mkdir trimming

~$ cd trimming

~$ nucmer --maxmatch –nosimplify \ ~/workspace/nanopolished_your_organism_name.fasta \ ~/workspace/nanopolished_your_organism_name.fasta

Note that “--maxmatch" and “--nosimplify" options are required for self- vs-self alignment. The first FASTA file (can be multifasta) is the “reference” while the second one is the “query”. As we want to see self-vs-self alignments, both are the same.

1. The output is an out.delta file. We can visualize this with ***Mummerplot*:**

~$ mummerplot out.delta

1. Obtain the exact coordinates of the repeats by looking at alignments near the start and end of the contig:

~$ show-coords -lrcTH out.delta

The output may look something like this:

1 5155 58347 63402 1895 1899 99.37 50400 50400 3.76 3.77 contig1 contig1

58347 63402 1 5155 1899 1895 99.37 50400 50400 3.77 3.76 contig1 contig1

Check if there are more repeats in the assembly, if the alignment is not 100% identical, and the lengths of the repeat sequences at the ends.

1. Trim to the sequences that you want to retain (*i.e.*, without repeats):

~$ samtools faidx \ ~/workspace/nanopolished_your_organism_name.fasta

~$ samtools faidx \ ~/workspace/nanopolished_your_organism_name.fasta \

contig1:1-58347 > final_your_organism_name.fasta

~$ cp final_your_organism_name.fasta ~/workspace/

“samtools faidx” is first used to index the genome. The second command extracts only the region you specify. Change “**contig1**” to the name of your chromosome (note: you cannot use the standard name of the *Canu* output) and the region “**1-58347**” to your specific requirements.

# Annotating your genome with Prokka

This final step uses an automated annotation pipeline to annotate the genes in your assembly. There are many tools available, but the easiest to use is ***Prokka***. *Prokka* uses prodigal to search for ORFs, and BLAST a “core” database from commonly found Uniprot proteins, and subsequently searches HMM-sensitive databases with HMMer3.

1. Use *Prokka* to annotate your genomes:

~$ prokka ~/workspace/final_your_organism_name.fasta \

--kingdom ‘Viruses’ --outdir prokka_output \

--prefix your_organism_name --cpus 1

Note that *Prokka* input is your final polished fasta file. “--kingdom" sets the kingdom of the organism. Change to ‘Bacteria’ if needed. Briefly, “--outdir" sets the name of the output directory, in this case prokka_output; “--prefix" sets the prefix name of the output, you can change that to what you want; “--cpus" sets the number of threads used, in this case 1.

In the output folder, you can find the **GFF (.gff)** and **Genbank (.gbk)** files that are created, which are the standard annotation file formats. Within these files, you can find each gene that is present in your genome, as well as tRNAs and rRNAs (for bacteria).

And that concludes it. You have now fully characterized a novel genome.

# References

1. Ewing B, Green P. Base-calling of automated sequencer traces using Phred. II. Error probabilities. Genome Res. 1998;8(3):186-94.
